# Growth differentiation factor 15 is positively associated with incidence of diabetes mellitus: the Malmö Diet and Cancer–Cardiovascular Cohort

**DOI:** 10.1007/s00125-018-4751-7

**Published:** 2018-10-22

**Authors:** Xue Bao, Yan Borné, Iram Faqir Muhammad, Jan Nilsson, Lars Lind, Olle Melander, Kaijun Niu, Marju Orho-Melander, Gunnar Engström

**Affiliations:** 1 Nutritional Epidemiology Institute and School of Public Health, Tianjin Medical University, Tianjin, China; 2 Department of Clinical Sciences, Lund University, Jan Waldenströms gata 35, CRC, hus 60 plan 13, 205 02 Malmö, Sweden; 3 Department of Medical Sciences, Uppsala University, Uppsala, Sweden

**Keywords:** Cohort analysis, Diabetes mellitus, Growth differentiation factor 15, Macrophage inhibitory cytokine-1

## Abstract

**Aims/hypothesis:**

Growth differentiation factor 15 (GDF-15) is an anti-inflammatory cytokine of the transforming growth factor-β superfamily. Circulating levels of GDF-15 are associated with hyperglycaemia among people with obesity or diabetes, but longitudinal evidence on the association between GDF-15 levels and diabetes risk is scarce. Our aim was to explore whether circulating levels of GDF-15 at baseline are positively associated with future diabetes incidence in a middle-aged urban population.

**Methods:**

Between 1991 and 1994, baseline fasting plasma GDF-15 levels were measured in 4360 individuals without diabetes (mean age 57.4 ± 5.96 years, 38.6% men) who were participants in the Malmö Diet and Cancer–Cardiovascular Cohort. After a follow-up of 19.0 ± 5.16 years (mean ± SD), Cox proportional hazards regression analysis was used for the study of the relationship between baseline GDF-15 and incident diabetes, with adjustment for established confounders. A sensitivity analysis included further adjustment for levels of C-reactive protein (CRP).

**Results:**

During the follow-up period, 621 individuals developed diabetes. The multivariate-adjusted HR for diabetes incidence was 1.43 (95% CI 1.11, 1.83; *p* for trend = 0.007) for the fourth compared with the first quartile of GDF-15, and was 1.17 (95% CI 1.07, 1.28; *p* < 0.001) per SD increase of GDF-15. If participants were grouped according to baseline fasting glucose, the association between GDF-15 and diabetes risk was only evident in the group without impaired fasting glucose (*n* = 3973). The association tended to be less significant with increasing age: multivariate-adjusted HRs for diabetes per SD increase of GDF-15 were 1.24 (95% CI 1.08, 1.42), 1.19 (95% CI 1.00, 1.41) and 1.04 (95% CI 0.89, 1.23) for participants aged ≤55, 56–60 (>55 and ≤60) and >60 years, respectively. With adjustment for levels of CRP, the HR per SD increase of GDF-15 (1.21, 95% CI 1.09, 1.35) was significant (*p* = 0.015), but the HR for the fourth compared with the first quartile of GDF-15 was not significant (HR 1.30; 95% CI 1.01, 1.67; *p* for trend = 0.061).

**Conclusions/interpretation:**

GDF-15 may be useful for identification of people with a risk of incident diabetes, especially if those people are ≤60 years old.

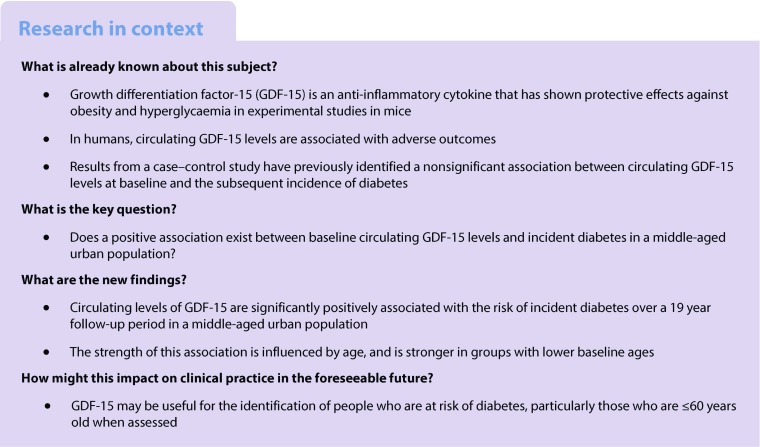



## Introduction

Growth differentiation factor 15 (GDF-15) is a divergent member of the transforming growth factor-β (TGF-β) cytokine superfamily [1, 2]. GDF-15 was initially identified as an autocrine inhibitor of macrophage activation and labelled macrophage inhibitory cytokine-1 [1], and its multiple roles in the regulation of cell proliferation, migration and maintenance of homeostasis were only discovered later [2]. In humans, GDF-15 is mainly expressed in macrophages and epithelial cells of various tissues [2], as well as in adipocytes. GDF-15 expression is positively associated with adiponectin production, which supports the proposal that GDF-15 is an adipokine [3]. Expression and secretion of GDF-15 can be dramatically enhanced by stimuli including injury, inflammation and malignancy [1, 4].

In experimental studies involving mice, GDF-15 has been found to control appetite [4, 5], reduce body weight and fat mass [4–7], increase thermogenesis, lipolysis and oxidative metabolism [6, 7], improve insulin sensitivity and glucose tolerance [5–7], and attenuate endothelial cell injury induced by high levels of glucose [8]. Because of these protective effects, GDF-15 has been considered to be beneficial for the prevention and treatment of obesity and hyperglycaemia [4–7]. However, in human studies, elevated GDF-15 concentrations are associated with adverse outcomes [9]. In individuals with obesity or diabetes, circulating levels of GDF-15 are higher than in unaffected individuals [10–12], and are positively correlated with blood concentrations of glucose, HbA_1c_, insulin resistance, C-reactive protein (CRP) and other cardiovascular risk factors [10, 11, 13]. GDF-15 levels are also associated with cardiovascular disease in individuals with established diabetes [14]. However, longitudinal data relating to the association between GDF-15 and diabetes risk are scarce. The only relevant study conducted to date was a prospective, nested case–control study [15], the results of which were that baseline circulating GDF-15 concentrations were higher in cases than in control participants, and had a nonsignificant positive association with the incidence of diabetes (age and sex-adjusted OR 1.21; 95% CI 0.997, 1.46; *p* = 0.054). In this study, the sample size was fairly small (180 cases of diabetes and 372 control participants) [15], and large cohort studies are still required to clarify whether circulating GDF-15 levels are associated with the incidence of diabetes. In the current study, our aim was to test this association in a middle-aged urban population in Sweden.

## Methods

### Participants

**Fig. 1:**
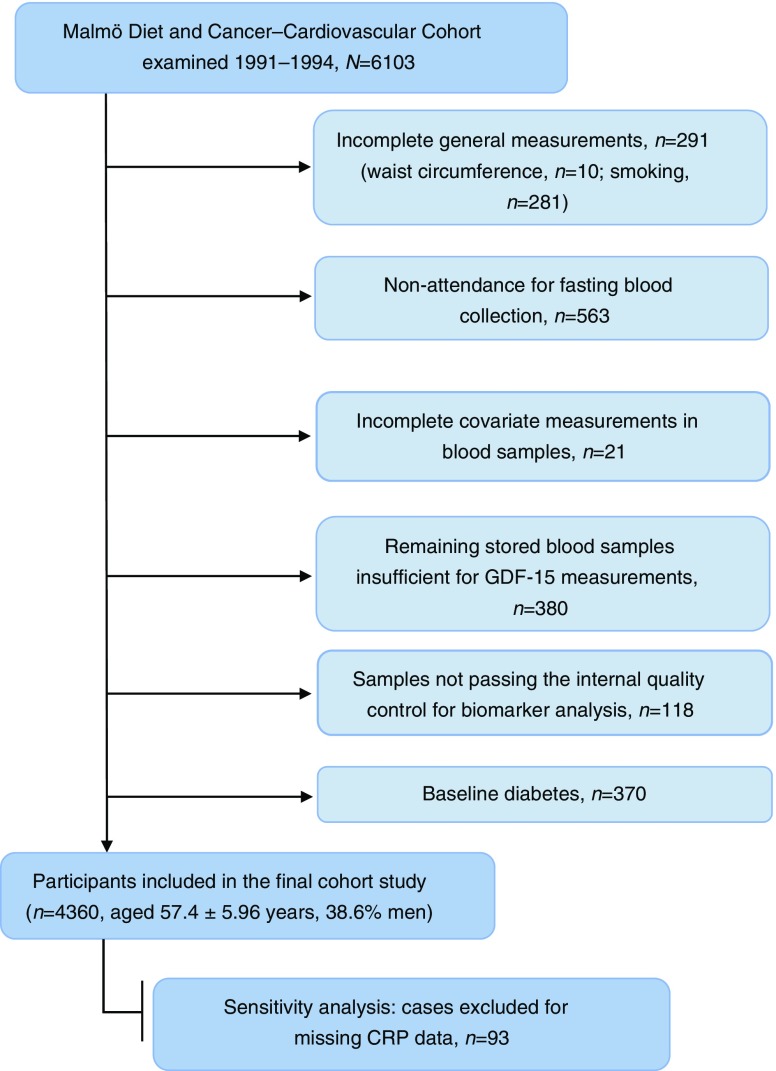
Study population flow chart

The Malmö Diet and Cancer (MDC) study is a large prospective cohort study that includes men and women from Malmö, a city in southern Sweden [16]. From 1991 to 1994, 6103 participants were randomly selected from the MDC study population and were invited to undergo additional examinations to study the epidemiology of carotid artery atherosclerosis. These individuals constituted the subcohort of the MDC cohort, the Malmö Diet and Cancer–Cardiovascular Cohort (MDC-CV) [17]. Among them, 291 were excluded on the basis of missing data on waist circumference or smoking. Of the remaining 5812 participants, 5249 attended a second appointment for fasting blood collection. Participants who had missing data on LDL-cholesterol or glucose (*n* = 21), or whose remaining stored blood samples were insufficient for GDF-15 measurement (*n* = 380), or who did not pass an internal quality control test for biomarker analysis (*n* = 118) were excluded, so that 4730 participants with complete data on GDF-15 and covariates remained in the study. Further exclusion of 193 individuals with self-reported diabetes or use of glucose-lowering medication, and 177 with fasting blood glucose ≥6.1 mmol/l (corresponding to a fasting plasma glucose cut-off of 7.0 mmol/l for the diagnosis of diabetes [18]), left 4360 participants for the cohort analysis (1682 men and 2678 women, age = 57.4 ± 5.96 years [mean ± SD]) (Fig. 1). Complete information on insulin and the HOMA index was available for 4336 participants. In sensitivity analyses, a further 93 individuals were excluded because CRP measurements were not available. All participants provided written informed consent. The study was approved by the Regional Ethical Review Board in Lund, Sweden (LU 51/90) (LU 2012/762) and was carried out in accordance with the Declaration of Helsinki.

### Baseline examinations

Anthropometric measurements were made at baseline using standard procedures. Waist circumference was determined midway between the lowest rib margin and iliac crest. Blood pressure was measured after individuals had rested for 10 min in a supine position. Information about current medication, smoking and alcohol consumption was obtained from a self-administered questionnaire. Participants were classified as non-smokers (former or never smokers) or current smokers (regular or occasional smokers). Men with alcohol intake >40 g per day and women with alcohol intake >30 g per day were classified as having a high level of alcohol consumption.

Blood samples were collected from the cubital vein after an overnight fast. Fresh plasma samples were used for measurement of levels of fasting blood glucose (mmol/l), according to standard procedures at the Department of Clinical Chemistry, University Hospital, Malmö. Insulin was measured by radioimmunoassay. The HOMA index was calculated as fasting insulin × fasting glucose/22.5 [17]. LDL-cholesterol concentrations (mmol/l) were estimated using the Friedewald formula [19]. Measurements of GDF-15 and CRP (nmol/l) used fasting plasma samples that had been stored at −80°C immediately after collection. CRP was analysed with the Tina-quant CRP latex assay (Roche Diagnostics, Basel, Switzerland). GDF-15 was measured by the SciLifeLab analysis service (Uppsala, Sweden) using the Proseek Multiplex CVD I ^96 × 96^ reagent kit (Olink Proteomics, Uppsala, Sweden) [20]. The high-specificity Proseek assay uses proximity extension technology [21, 22], so that antibody binding of antigens (such as GDF-15) brings oligonucleotide pairs into proximity, enabling PCR amplification of a reporter sequence. For GDF-15, the lower and upper limits of quantification were 15.3 and 62,500 pg/ml, respectively. Intra-assay variation was 9%, which was the mean coefficient of variation for seven individual samples within each of nine separate runs during validation. Inter-assay variation was 11%, which was the mean coefficient of variation for the same seven individual samples between the nine separate runs during validation. Raw Proseek data underwent a pre-processing normalisation procedure. For each data point (corresponding to PCR amplification resulting from binding to a specific protein in one sample well), normalisation for technical variation (such as pipetting differences) was performed by subtraction of the quantification cycle (C_q_) value in that well for the extension control (an antibody linked to a pair of oligonucleotides, resulting in antigen-independent PCR amplification). To control for variation between plates, a standard sample containing goat IgG conjugated to each of the 92 oligonucleotide pairs of the assay (the inter-plate control [IPC]) was added to three wells of each plate. Normalisation between runs was performed by subtracting the median IPC C_q_ from all the extension control-adjusted values on a plate, resulting in normalised protein expression (NPX, log_2_ scale) values. The NPX values were finally adjusted to give a background level (from negative controls) of around zero. Levels of plasma GDF-15 measured by the Proseek assay correlated closely with those measured with an electrochemiluminescence immunoassay (Roche Diagnostics, Mannheim, Germany) [23] in a sample of 987 men and women from a different population to the cohort in the current study (*r* = 0.89 [L. Lind, unpublished data]).

### Outcomes

All participants without a previous history of diabetes were followed until incident diabetes, emigration from Sweden, death or the end of follow-up (31 December 2014), whichever came first. Information on new diabetes cases was retrieved from both local and national registers [24], consisting of the Swedish National Diabetes Register (NDR), the regional Diabetes 2000 register of the Scania region, the Malmö HbA_1c_ register (MHR), the Swedish inpatient register, the Swedish outpatient register and the nationwide Swedish drug prescription register. In the Swedish NDR and the Diabetes 2000 register, new cases of diabetes were diagnosed according to established criteria (fasting plasma glucose concentration ≥ 7.0 mmol/l with two repeated tests on separate occasions). In MHR, individuals were considered to have developed diabetes if they had at least two HbA_1c_ recordings ≥42 mmol/mol (6.0%) with the Swedish Mono-S standardisation system (corresponding to 53 mmol/mol [7.0%] according to the US National Glycohemoglobin Standardization Program) after the baseline examination. In the Swedish inpatient and outpatient registers, diabetes was diagnosed by a senior physician. In the nationwide prescription register, a filled prescription of insulin or glucose-lowering medications (ATC-code A10) was required for diagnosis of diabetes.

### Statistical analyses

Because the distribution of values was skewed, CRP measurements were logarithmically transformed before analyses. Participants were divided into quartiles according to GDF-15 concentrations, with a stable proportion of men and women across quartiles. To describe baseline characteristics of participants in each quartile, continuous variables are presented as means ± SD (or medians [25–75%] for skewed distributions), and categorical variables are presented as percentages. Differences in characteristics across GDF-15 quartiles were determined by analysis of variance for continuous variables and logistic regression for categorical variables. Multiple linear regression including other known covariates was used for identification of factors independently associated with GDF-15 concentration. The incidence of diabetes across GDF-15 quartiles was plotted with the Kaplan–Meier curve. Cox proportional hazards regression was used for assessment of the associations between baseline GDF-15 quartiles or SD increments of GDF-15 and incident diabetes, with ‘time in study’ as the timescale. In a sensitivity analysis, age was also used as the timescale. The analyses were adjusted for potential confounders including age, sex, waist circumference, LDL-cholesterol, fasting glucose, systolic blood pressure, anti-hypertensive medication, smoking and alcohol consumption. A sensitivity analysis was also adjusted for CRP. In other sensitivity analyses waist circumference was replaced with body mass index or waist:hip ratio, and adjustment was made for physical activity and total energy intake (see [25] for detailed measurement methods).

Possible interactions between GDF-15 levels and risk factors, with respect to incidence of diabetes, were explored by introduction of interaction terms in the multivariate model (one at a time). Restricted cubic spline functions [26, 27] were incorporated into the Cox model to explore the shape of the association between GDF-15 and diabetes in adjusted analyses, with knots placed at 20%, 40%, 60% and 80% of GDF-15 concentration. Similarly, possible time-dependent effects were estimated with knots placed at 5, 10, 15 and 20 years of follow-up. If (post hoc) threshold or time-dependent effects were detected, risk was estimated by fitting piecewise linear models [26] or sub-distribution hazards models with time-dependent effects [28], respectively. A competing-risks model was applied to investigate the association between GDF-15 and incidence of diabetes, with deaths from causes other than diabetes used as competing events [28].

HRs and corresponding 95% CIs were calculated. A two-tailed *p* < 0.05 was considered to be statistically significant. All analyses were performed using the Statistical Analysis System version 9.3 for Windows (SAS Institute, Cary, NC, USA).

## Results

The study population included 4360 individuals aged 57.4 ± 5.96 years (mean ± SD). During a follow-up period of 19.0 ± 5.16 years, 621 participants developed diabetes. The incidence rate was 7.50 per 1000 person-years. Participants who developed diabetes during follow-up had higher baseline GDF-15 levels than participants who were free of diabetes at the end of follow-up (*p* < 0.001, data not shown).

Baseline characteristics of participants stratified by quartiles of GDF-15 are shown in Table 1. Levels of almost all risk factors showed significant trends, increasing from the lowest to the highest GDF-15 quartile, with the exception of high-level alcohol consumption, which did not differ statistically across the groups. Multiple linear regression revealed that age was the factor with the strongest independent association with baseline GDF-15 concentrations (standardised β = 0.343, *p* < 0.001). Smoking, CRP, male sex and systolic blood pressure were also positively correlated with GDF-15 in the multiple linear regression model (standardised β = 0.231, 0.151, 0.083 and 0.034, respectively; all *p* < 0.05). Waist circumference, high-level alcohol consumption, LDL-cholesterol, fasting glucose and anti-hypertensive medication were not significantly associated with GDF15 in this multiple linear regression model (all *p* ≥ 0.05).

**Table 1:** Characteristics of individuals across quartiles (Q1–Q4) of GDF-15 (*N* = 4360)

Characteristics	GDF-15 quartiles				*p* for trend^a^
	Q1	Q2	Q3	Q4	
*n*	1091	1089	1091	1089	–
GDF-15 range (arbitrary units)^b^	6.09–8.44	8.34–8.79	8.66–9.16	9.01–12.30	–
Age (years)	54.5 ± 5.53	56.6 ± 5.71	58.4 ± 5.54	59.9 ± 5.60	<0.001
Fasting glucose (mmol/l)	4.84 ± 0.43	4.86 ± 0.46	4.92 ± 0.45	4.93 ± 0.47	<0.001
HOMA-IR^c^ (*N* = 4336)	1.23 (0.82, 1.71)	1.28 (0.80, 1.88)	1.39 (0.89, 1.99)	1.46 (0.92, 2.22)	0.020
CRP (nmol/l)^c^ (*N* = 4267)	9.52 (4.76, 18.1)	10.5 (5.71, 21.0)	12.4 (6.67, 26.7)	19.0 (8.57, 38.1)	<0.001
Systolic blood pressure (mmHg)	135.7 ± 16.5	139.5 ± 18.6	141.9 ± 19.2	144.0 ± 19.0	<0.001
Diastolic blood pressure (mmHg)	85.4 ± 8.51	86.3 ± 9.18	86.9 ± 9.44	87.1 ± 9.47	<0.001
Waist circumference (cm)	81.4 ± 11.5	81.6 ± 11.8	82.8 ± 12.5	84.2 ± 12.8	<0.001
Body mass index (kg/m^2^)	25.2 ± 3.54	25.2 ± 3.47	25.6 ± 3.85	25.8 ± 3.98	0.007
LDL-cholesterol (mmol/l)	4.09 ± 0.94	4.07 ± 0.94	4.24 ± 0.96	4.27 ± 1.04	<0.001
Smokers, *n* (%)	105 (9.62)	185 (17.0)	268 (24.6)	389 (35.7)	<0.001
High level of alcohol consumption, *n* (%)	43 (3.67)	34 (3.12)	33 (3.03)	34 (3.12)	0.471
Anti-hypertensive medication, *n* (%)	119 (10.9)	144 (13.2)	177 (16.2)	204 (18.7)	<0.001
Statin medication, *n* (%)	5 (0.46)	18 (1.65)	18 (1.65)	18 (1.65)	0.02

Values are expressed as mean ± SD or *n* (%), unless otherwise indicated

^a^Analysis of variance or logistic regression analysis

^b^GDF-15 is expressed as NPX values on a log_2_ scale; ranges of GDF-15 concentration across quartiles were 6.09–8.44, 8.44–8.79, 8.79–9.16 and 9.16–11.5 for males, and were 6.78–8.34, 8.34–8.66, 8.66–9.01 and 9.01–12.30 for females

^c^Values expressed as median (25–75%)

**Fig. 2:**
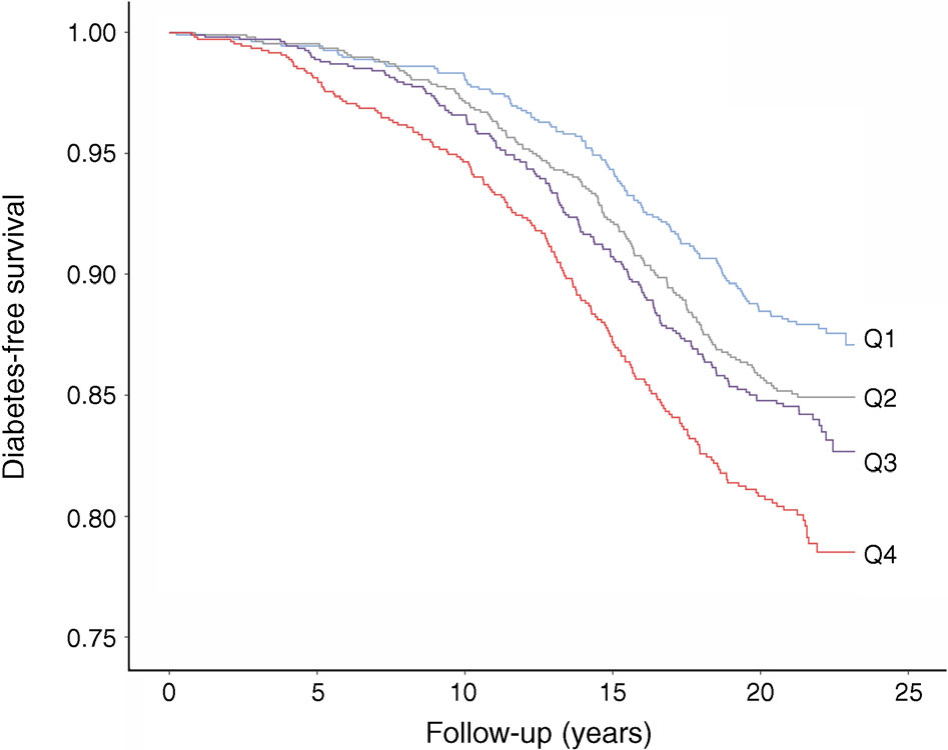
Diabetes-free survival across quartiles (Q1–Q4) of baseline GDF-15 concentration

Diabetes-free survival was shorter among participants with high baseline GDF-15 concentrations than in those with low baseline GDF-15 (Fig. 2). The crude and adjusted relationships between GDF-15 quartiles and incidence of diabetes are shown in Table 2. The risk of developing diabetes increased with increasing quartiles of GDF-15. After multivariate adjustment (Table 2, Model 3), the HR for diabetes in the fourth compared with the first quartile of GDF-15 was 1.43 (95% CI 1.11, 1.83; *p* for trend = 0.007). The multivariate-adjusted HR for diabetes per SD increase of GDF-15 was 1.17 (95% CI 1.07, 1.28; *p* < 0.001). Further adjustment for CRP attenuated the association between GDF-15 quartiles and diabetes risk. The HR for diabetes in the fourth compared with the first quartile of GDF-15 was 1.30 (95% CI 1.01, 1.67; *p* for trend = 0.061), but the association between GDF-15 (in SD units) and diabetes remained significant (HR 1.12; 95% CI 1.02, 1.23; *p =* 0.015). Results changed only marginally when body mass index (or waist:hip ratio), physical activity or total energy intake were taken into consideration in multivariate analyses (data not shown).

**Table 2:** Relationships between circulating levels of GDF-15 (per SD increase or by quartiles [Q1–Q4]) and incidence of diabetes (*N* = 4360)

	Per SD increase of GDF-15	*p*	GDF-15 quartiles				*p* for trend^a^
			Q1	Q2	Q3	Q4	
*n*		–	1091	1089	1091	1089	–
Incidence, *n*		–	125	152	159	185	–
Incidence, *n* (per 1000 person-years)		–	5.68	7.08	7.70	9.89	–
Model 1^b^_,_ HR (95% CI)	1.28 (1.19, 1.39)	<0.001	Reference	1.25 (0.99, 1.59)	1.39 (1.10, 1.75)	1.84 (1.47, 2.31)	<0.001
Model 2^c^_,_ HR (95% CI)	1.22 (1.12, 1.33)	<0.001	Reference	1.27 (1.00, 1.61)	1.33 (1.04, 1.69)	1.66 (1.30, 2.11)	<0.001
Model 3^d^_,_ HR (95% CI)	1.17 (1.07, 1.28)	<0.001	Reference	1.17 (0.92, 1.49)	1.16 (0.91, 1.49)	1.43 (1.11, 1.83)	0.007

^a^Analysis by Cox proportional hazards model

^b^Crude model

^c^Adjusted for sex, age and waist circumference

^d^Adjusted for sex, age, waist circumference, LDL-cholesterol, fasting glucose, systolic blood pressure, anti-hypertensive medication, smoking and alcohol consumption

**Fig. 3:**
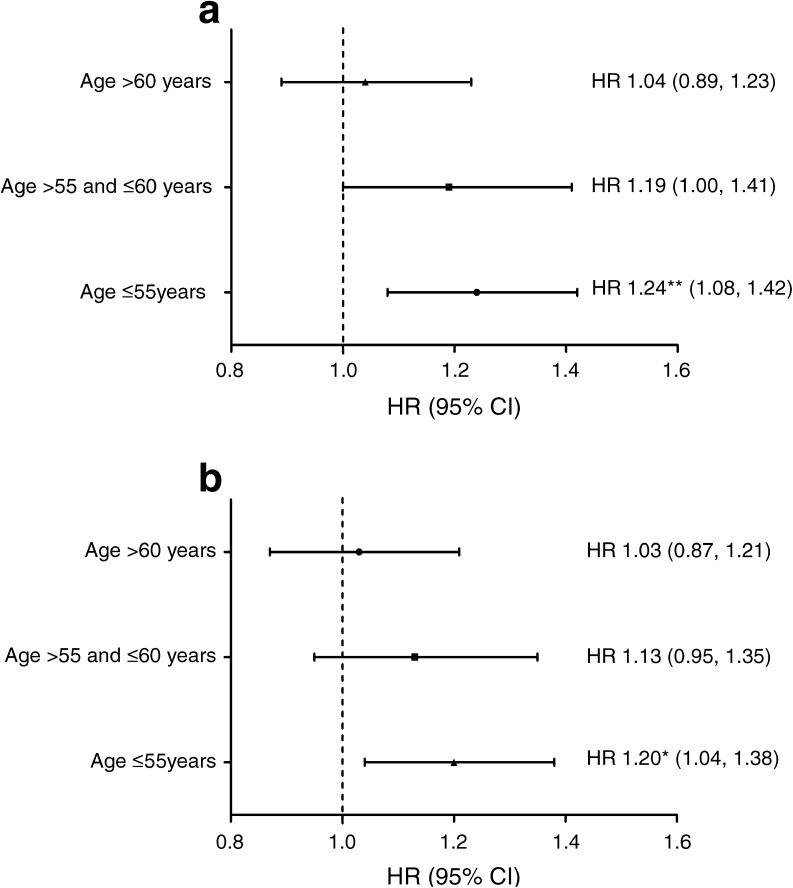
Association of baseline levels of GDF-15 with incident diabetes over long-term follow-up. (**a**) Association of GDF-15 (per SD increase) with diabetes risk, divided by age groups, adjusted for sex, waist circumference, LDL-cholesterol, fasting glucose, systolic blood pressure, anti-hypertensive medication, smoking and alcohol consumption. (**b**) Association of GDF-15 (per SD increase) with diabetes risk, divided by age groups, additionally adjusted for CRP. *p<0.05, **p<0.01

We observed significant interaction between GDF-15 and age in the association with incident diabetes (*p* for interaction <0.001). Therefore, participants were divided into three age groups: younger (≤55 years old, *n* = 1758), intermediate (>55 and ≤ 60 years old, *n* = 1014) and older (>60 years old, *n* = 1588). The association between GDF-15 and diabetes tended to be less significant with increasing age (Fig. 3). A moderate interaction was also observed between GDF-15 and fasting glucose (*p* for interaction = 0.04). Fasting blood glucose was stratified by a cut-off value of 5.6 mmol/l (corresponding to a cut-off of 6.1 mmol/l for definition of impaired fasting glucose using plasma [18]). Significant association between GDF-15 and diabetes risk was found only in participants with fasting glucose <5.6 mmol/l (*n* = 3973). In this group, the adjusted HR for diabetes per SD increase in GDF-15 was 1.21 (95% CI 1.09, 1.35; *p* < 0.001), and with additional adjustment for CRP this HR was 1.16 (95% CI 1.04, 1.29; *p* = 0.009). No interaction between GDF-15 and any other covariate was identified.

The association of GDF-15 with diabetes risk was generally linear (*p* for non-linearity = 0.363), and was not time dependent (*p* for time dependency = 0.051). However, because this *p* value was close to being significant, HRs at different time points were calculated, and a slightly decreasing effect of GDF-15 with time was observed (adjusted HRs for diabetes per SD increase in GDF-15 were 1.28 [95% CI 1.13, 1.44] and 1.20 [1.09, 1.33], respectively, at 10 and 20 years of follow-up). When death from causes other than diabetes was accounted for as a competing event, the association between GDF-15 and the risk of diabetes was attenuated, but remained significant. In a multivariate competing-risk model, HRs for diabetes were 1.33 (95% CI 1.03, 1.71; *p* for trend = 0.049) for the fourth vs the first quartile of GDF-15, and 1.10 (95% CI 1.00, 1.21; *p* = 0.042) per SD increase in GDF-15.

## Discussion

The results of this cohort study showed that, in an urban population, circulating levels of GDF-15 were positively associated with the risk of incident diabetes, after controlling for traditional risk factors. The associations were evident in people without impaired fasting glucose and were considerably stronger in people ≤60 years old than in those >60 years old at baseline.

Results from previous studies have shown that individuals with obesity or diabetes have significantly higher GDF-15 concentrations than control participants [10–12]. In addition, GDF-15 levels are higher in obese individuals with (than in those without) impaired glucose tolerance [13], and are also higher in people with diabetes than in those without it [10, 11]. Our results are supported by those of a nested case–control study involving 180 participants with diabetes and 372 control participants) [15], which demonstrated that circulating GDF-15 concentrations are higher in individuals from the diabetes group than in those from the control group, even before diagnosis. However, this evidence was inconclusive [15], whereas our results demonstrated that the longitudinal association between GDF-15 and diabetes remained significant even after adjustment. Our study had a larger sample size than the previous study, and greater statistical power for the detection of associations. Results from another previous cohort study [13] demonstrated that baseline GDF-15 is associated with future insulin resistance and impaired glucose control. The study population consisted of obese individuals who were enrolled in a clinical trial, rather than a sample of the general population. Moreover, the small size of the study population meant that it was not possible to explore the association between GDF-15 and incidence of diabetes [13]. By contrast, our results were derived from a large-scale cohort study, and showed that baseline GDF-15 was positively associated with the incidence of diabetes in the 19 year follow-up period.

GDF-15 is an anti-inflammatory cytokine, and other anti-inflammatory markers (such as TGF-β1) are also known to be elevated before the onset of diabetes [29, 30]. Like GDF-15, TGF-β1 is a member of the TGF-β cytokine superfamily [30]. It has been suggested that upregulation of GDF-15 occurs in response to inflammation that precedes diabetes, but that this upregulation is not sufficient to compensate for the chronic low-grade inflammation [31], which leads to metabolic disorders such as β cell dysfunction and insulin resistance [31, 32]. Another link between GDF-15 and hyperglycaemia might involve activation of the transcription factor p53. In adipose tissue from both mice and humans, obesity-induced p53 activation contributes to inflammation and insulin resistance [33]. Results from studies carried out in vitro have shown that upregulation of GDF-15 expression occurs in a p53-dependent manner [8, 34]. Thus, GDF-15 may serve as a surrogate marker of p53 activation in the pathogenesis of obesity and hyperglycaemia.

Our results showed that GDF-15 was positively correlated with various cardiovascular risk factors, which is consistent with the findings of previous studies [10, 11, 13, 15, 35]. Age, smoking and CRP are important risk factors that were independently associated with GDF-15 levels. The anti-inflammatory nature of GDF-15 suggests a mechanism in which its expression may be increased as a compensatory response to inflammation (as depicted by CRP) or proinflammatory stimuli (such as smoking). Of the three risk factors, age had the strongest correlation with GDF-15, which is in accordance with previous findings [11, 13, 36]. Age may be associated with GDF-15 expression via both physiological and pathological processes. Evidence suggests that GDF-15 is a potential biomarker for ageing and age-related cognitive decline [37, 38]. An elevated concentration of circulating GDF-15 could reflect mitochondrial dysfunction, which is a hallmark of ageing, and which contributes to the pathogenesis of various age-related disorders [38, 39]. In addition, oxidative stress, inflammation, protein glycation, cellular senescence and hormonal deregulation may develop with age [40]. Such dysregulations may contribute to higher GDF-15 expression, possibly via the transcription factors early growth response protein-1 or p53 [8, 34, 41]. The complexity of the relationship between ageing and GDF-15 expression could at least partly explain why the strength of the association that we observed between GDF-15 and diabetes risk changed with age and with follow-up duration. Further studies are required to clarify the exact role of ageing in the association between GDF-15 and diabetes risk.

In our study, no significant association between GDF-15 and diabetes risk was detected in participants with impaired fasting glucose. One possible explanation for this observation is that our study had insufficient power to detect an association in this group because of the limited sample size (*n* = 387). Another possibility is that in this group, compensatory secretion of GDF-15 might have reached a maximum in response to pre-existing metabolic disturbance.

Strengths of this study include its prospective design, the large sample (which was randomly selected from the general urban population) and the long-term follow-up with a high follow-up rate (>99.0%) [16]. Some limitations also need to be considered. GDF-15 was measured in arbitrary units, on the basis of real-time PCR quantification, so the concentrations cannot be directly compared with results derived from other assays. However, the study cohort was drawn from the general urban population, so the concentrations of GDF-15 could, by definition, be regarded as normal [42]. Furthermore, correlation was found to be high (*r* = 0.89 [L. Lind, unpublished data]) between GDF-15 levels measured by the Proseek proximity extension method and by an electrochemiluminescence immunoassay in a sample of 987 individuals from a different population to the cohort in the current study. Another potential limitation is that measurement of GDF-15 was conducted only once, using frozen blood samples stored at −80°C for more than a decade. The long-term stability of GDF-15 in these samples is not known, but GDF-15 levels in stored baseline samples have previously been found to correlate strongly with levels in samples taken at 5 year follow-up, when all samples were analysed at the same time [43]. In addition, in a study of similar design to ours, baseline GDF-15 levels measured in samples frozen for up to 18 years had a predictive ability for adverse outcomes [44]. These findings suggest that such samples are sufficiently stable. In our study, we did not distinguish diabetes types. However, the study population included only middle-aged adults, and because symptoms of type 1 diabetes usually appear in early life, individuals with type 1 diabetes should have been thoroughly excluded at baseline as prevalent cases. All incident cases in the present study were very likely to be type 2 diabetes.

Type 2 diabetes is often asymptomatic during the initial stages, and may remain unrecognised for several years [45]. Our findings support the view that metabolic manifestations and corresponding compensatory responses can precede the onset of diabetes by many years. In particular, the results of sub-analyses implied that the compensatory reactions might be more sensitive in people who are relatively young (≤60 years old) or who have normal fasting glucose, suggesting that these might be the most appropriate populations in which to use GDF-15 to evaluate diabetes risk. Even though the observed association remained significant after extensive adjustments, unknown residual confounding could exist, and further studies are still needed to provide confirmation of the relationship. As one element in that relationship, the role of ageing may be better understood if specific ageing-related variables such as telomere length [46] or mitochondrial function [38] are taken into consideration.

In conclusion, the results of this study identified a positive association between baseline levels of circulating GDF-15 and diabetes risk in a 19 year follow-up period. The strength of this association was influenced by age at baseline. GDF-15 may be useful for identification of people with a risk of incident diabetes, especially if those people are ≤60 years old.

## Data Availability

The data that support the findings of this study are available from Lund University, but restrictions apply to the availability of these data, which were used under licence for the current study, and so are not publicly available. Data are, however, available from the corresponding author (XB) upon reasonable request and with permission of Lund University.

## Abbreviations

CRP: C-reactive protein
GDF-15: Growth differentiation factor 15
IPC: Inter-plate control
MDC: Malmö Diet and Cancer
MHR: Malmö HbA_1c_ register
NDR: National Diabetes Register
NPX: Normalised Protein Expression Unit
PEA: Proximity extension assay
